# Cervical carcinoma risk associate with genetic polymorphisms of *NEIL2* gene in Chinese population and its significance as predictive biomarker

**DOI:** 10.1038/s41598-020-62040-9

**Published:** 2020-03-20

**Authors:** Feng Ye, Jia Liu, Hanzhi Wang, Xiaojing Chen, Qi Cheng, Huaizeng Chen

**Affiliations:** 1 Central Laboratory of Women’s Hospital, School of Medicine, Zhejiang University, Hangzhou City, Zhejiang Province China; 2 Women’s Reproductive Health Key Laboratory of Zhejiang Province, Women’s Hospital, School of Medicine, Zhejiang University, Hangzhou City, Zhejiang Province China; 3 Department of Gynecology, Women’s Hospital, School of Medicine, Zhejiang University, Hangzhou City, Zhejiang Province China

**Keywords:** Predictive markers, Cancer genetics, Risk factors

## Abstract

Genetic polymorphisms of *NEIL1* and *NEIL2* maybe change protein function, and increased carcinogenesis. In this study, seven *NEIL1* SNPs and three *NEIL2* SNPs were selected. 400 CSCCs, 400 CIN III, and 1200 normal healthy controls were genotyped by mismatch amplification PCR. mRNA and protein expression of *NEIL*2 was measured in 92 freshly-obtained CSCC tumor tissues. The association between homozygote CC genotype of *NEIL2* rs804270 with susceptible risk was gradually increased in CIN III (OR = 1.44) and CSCC (OR = 2.22). Carriers of C-allele (GC + CC) at rs804270 had a high risk of CSCC (OR = 1.46). The heterozygote GT genotype of rs8191664 was also closely related to the higher risk of CINIII (OR = 1.59) and CSCC (OR = 2.54). Carriers of T-allele (GT + TT) at rs8191664 had a high risk for CIN III (OR = 1.55) and CSCC (OR = 2.34). The genotypes of *NEIL2* rs804270 (G/C) and rs8191664 (G/T) that were related to the higher risk for CIN III were CC-GG (OR = 1.42) and CC-GT (OR = 2.07). More notably, there was a greater risk for CSCC with the GC-GT (OR = 1.91), CC-GG (OR = 1.67), and CC-GT (OR = 6.18) genotypes. *NEIL2* mRNA expression in CSCCs with the rs804270-CC genotype was lower expression than those in CSCCs with the rs804270-GG and rs804270-GC genotypes. Similarly, *NEIL*2 protein expression was significantly decreased in CSCCs with the rs804270-CC genotype. In summary, the two genetic polymorphisms (rs804270 and rs8191664) of *NEIL*2 gene were significantly associated to the increased susceptibility of CIN III or CSCC. This increased susceptibility maybe due to altered *NEIL*2 repair activity through altered protein expression, or changed structure of the functional domain. The genotypes of GC-GT, CC-GG, and CC-GT of rs804270 and rs8191664 of *NEIL2* gene could act as a genetic predictive biomarker of susceptibility to CIN III and CSCC.

## Introduction

Globally, cervical carcinoma is the fourth most common malignant cancer in women with approximately 500,000 new cases and almost 300,000 deaths each year^1^. Cervical carcinoma is also the third leading cause of cancer-related death in women; this is very worrying because the incidence of this disease is rising^2^. The primary etiological factor is the infection by the high risk human papilloma virus (HR-HPV)^3^. Aside from breast cancer, cervical carcinoma has become the most common form of cancer in Chinese women (with an incidence of 98.9 patients per 100,000 of the Chinese population). The mortality rate from cervical carcinoma has increased to 30.5 per 100,000^4^. However, while 80% of women will become infected with HPV during their lifetime, only a small number will develop malignant cervical carcinoma^5^. Epidemiological evidence has confirmed that a range of genetic variations are associated with the risk of cervical carcinoma^6^.

Previous research, including two genome-wide studies, have identified loci that are genetically susceptible and genetic polymorphisms that are closely related to the occurrence of cervical carcinoma^6–8^. However, these genetic polymorphisms account for only a small part of the genetic susceptibility to cervical cancer. Therefore, more comprehensive and in-depth genetic study is needed to further understand the genetic risk factors for cervical carcinoma.

The integrity and genetic stability of the genome are maintained by a variety of DNA repair systems in order to combat environmental attacks, replication mistakes and cumulative geriatric degeneration. There are five major DNA repair mechanisms in the human genome that are used to repair damaged DNA, including direct reversal, nucleotide excision repair, base excision repair, mismatch repair and recombination repair; previous studies have shown that more than 100 genes are involved in these mechanisms^9^.

During mammalian cell replication, the repair of damaged DNA caused by reactive oxygen species (ROS) is mainly performed by a group of DNA glycosylases, including two DNA glycosylase genes, *NEIL1* and *NEIL2*. *NEIL1* and *NEIL2* can protect normal somatic cells from radiation damage; if these genes are subject to functional genetic variation, then it is likely that their normal protein function may be changed, eventually leading to a change in cell fate and increased carcinogenic potential^10–13^. Several reports have shown that genetic variations in *NEIL1* and *NEIL2* are significantly associated with susceptibility to solid malignant tumors, such as oropharyngeal cancer^14^, gastric cancer^15^, bladder cancer^16^ and colorectal adenoma^17^. However, from these published studies, we found that the association analysis data of single nucleotide polymorphisms (SNPs) of these two genes with cancer risk are not comprehensive, and their protein expression and functional activity have not been generally studied. In addition, the correlation between SNP loci in *NEIL1* and *NEIL2* and susceptibility to cervical carcinoma has not been studied so far.

Therefore, in our large-sample population-based study, we selected seven SNP loci in *NEIL1* and three SNP loci in *NEIL2*, then investigated their genotype frequency in 400 cervical squamous cell carcinomas (CSCC), 400 cervical intraepithelial neoplasias (CIN III) and 1200 normal healthy controls, and analyzed the association between these SNPs in the *NEIL1* and *NEIL2* genes and susceptibility to CSCC and CIN III. Furthermore, we also detected the expression of the *NEIL2* gene in different genotypes of cervical cancer cells at the mRNA and protein level to investigate the relationship between SNP genotypes and gene expression. The purpose of this study was to better understand the potential role of specific SNP genotypes in the carcinogenesis of CSCC.

## Results

### The relationship between genetic polymorphisms in NEIL1 and NEIL2 and the risk of CIN III or CSCC

As show in Table 1, the genotype or allele frequencies of genetic polymorphisms in *NEIL1* and *NEIL2* were rs4462560, rs7182283, rs7402844, rs5745920, rs8030014, rs11634109 and rs79244935 for *NEIL1*, and rs804270, rs8191613 and rs8191664 for *NEIL2*. Hardy-Weinberg Equilibrium(HWE) test was performed for all of SNP alleles in normal healthy control group(Shown in Table S2**)**, the P value of HWE analysis of some loci is less than 0.05, which indicates that the specific genotypes of these loci have certain enrichment in Chinese population. Combined with the statistical results of Tables 1 and 2, we believe that the enrichment of some loci in normal healthy control group does not affect the comparison of genotype frequency between disease group and normal healthy control group.

**Table 1:** Association between NEIL1 and NEIL2 genetic variants and the risk of CIN III and CSCCs.

Genotypes	Normal healthy controls (N = 1200)		CIN III (N = 400)		adjusted OR* (95% CI)	*P*^*#*^	*Pa*	CSCCs (N = 400)		adjusted OR* (95% CI)	*P*^*#*^	*Pa*	*χ*^2^	P*
	N	%	N	%				N	%					
***NEIL1***														
**rs4462560**													0.040	0.980
CC	433	36.1	142	35.5	1.00(ref)			131	32.8	1.00(ref)				
CG	547	45.6	189	47.3	1.05(0.82–1.36)	0.684	0.977	207	51.8	1.25(0.97–1.61)	0.082	0.328		
GG	220	18.3	69	17.3	0.96(0.69–1.33)	0.791	1.055	62	15.5	0.93(0.66–1.31)	0.685	0.761		
CG + GG	767	63.9	258	64.5	1.03(0.81–1.30)	0.833	0.833	269	67.3	1.16(0.91–1.47)	0.227	0.757		
Allelic frequency														
Allele C	1413	58.9	473	59.1	1.00(ref)			469	58.6	1.00(ref)				
Allele G	987	41.1	327	40.9	0.99(0.84–1.17)	0.901	0.901	331	41.4	1.01(0.86–1.19)	0.901	1.001		
**rs7182283**													0.647	0.724
GG	356	29.7	124	31.0	1.00(ref)			109	27.3	1.00(ref)				
GT	627	52.3	201	50.3	0.92(0.71–1.19)	0.530	0.815	217	54.3	1.13(0.87–1.47)	0.364	0.910		
TT	217	18.1	75	18.8	0.99(0.71–1.38)	0.964	0.964	74	18.5	1.11(0.79–1.57)	0.535	0.713		
GT + TT	844	70.3	276	69.0	0.94(0.74–1.20)	0.614	0.682	291	72.8	1.13(0.88–1.45)	0.357	0.714		
Allelic frequency														
Allele G	1339	55.8	449	56.1	1.00(ref)			435	54.4	1.00(ref)				
Allele T	1,061	44.2	351	43.9	0.99(0.84–1.16)	0.869	1.086	365	45.6	1.06(0.90–1.24)	0.485	0.606		
**rs7402844**													4.454	0.108
GG	527	43.9	182	45.5	1.00(ref)			167	41.8	1.00(ref)				
GC	402	33.5	137	34.3	0.99(0.76–1.28)	0.919	1.021	121	30.3	0.95(0.73–1.24)	0.706	0.706		
CC	271	22.6	81	20.3	0.87(0.64–1.17)	0.345	0.863	112	28.0	1.30(0.99–1.73)	0.064	0.427		
GC + CC	673	56.1	218	54.5	0.94(0.75–1.78)	0.581	0.726	233	58.3	1.09(0.87–1.37)	0.449	0.561		
Allelic frequency														
Allele G	1456	60.7	501	62.6	1.00(ref)			455	56.9	1.00(ref)				
Allele C	944	39.3	299	37.4	0.92(0.78–1.09)	0.325	0.650	345	43.1	1.17(0.99–1.38)	0.058	0.193		
**rs5745920**													2.389	0.303
CC	374	31.2	114	28.5	1.00(ref)			123	30.8	1.00(ref)				
CT	664	55.3	227	56.8	1.12(0.87–1.45)	0.384	0.768	204	51.0	0.93(0.72–1.21)	0.604	0.755		
TT	162	13.5	59	14.8	1.20(0.82–1.72)	0.338	0.966	73	18.3	1.37(0.97–1.93)	0.072	0.360		
CT + TT	826	68.8	286	71.5	1.14(0.89–1.46)	0.316	0.632	277	69.3	1.02(0.80–1.30)	0.876	0.876		
Allelic frequency														
Allele C	1412	58.8	455	56.9	1.00(ref)			450	56.3	1.00(ref)				
Allele T	988	41.2	345	43.1	1.08(0.92–1.27)	0.331	0.552	350	43.8	1.11(0.95–1.31)	0.200	0.500		
**rs8030014**													0.631	0.729
AA	323	26.9	122	30.5	1.00(ref)			118	29.5	1.00(ref)				
AG	664	55.3	195	48.8	0.78(0.60–1.01)	0.060	0.400	213	53.3	0.88(0.68–1.14)	0.329	1.097		
GG	213	17.8	83	20.8	1.03(0.74–1.43)	0.852	1.065	69	17.3	0.89(0.63–1.25)	0.493	0.704		
AG + GG	877	73.1	278	69.5	0.84(0.66–1.08)	0.166	0.553	282	70.5	0.88(0.69–1.13)	0.317	0.793		
Allelic frequency														
Allele A	1310	54.6	439	54.9	1.00(ref)			449	56.1	1.00(ref)				
Allele G	1,090	45.4	361	45.1	0.99(0.84–1.16)	0.886	0.984	351	43.9	0.94(0.80–1.10)	0.448	0.640		
**rs11634109**													0.474	0.789
TT	993	82.8	341	85.3	1.00(ref)			333	83.3	1.00(ref)				
TC	197	16.4	52	13.0	0.77(0.55–1.07)	0.117	0.585	62	15.5	0.94(0.69–1.28)	0.689	0.725		
CC	10	0.8	7	1.8	2.04(0.77–5.40)	0.152	0.507	5	1.3	1.49(0.51–4.39)	0.469	0.853		
TC + CC	207	17.3	59	14.8	0.83(0.61–1.14)	0.245	0.613	67	16.8	0.97(0.71–1.31)	0.818	0.909		
Allelic frequency														
Allele T	2183	91.0	734	91.8	1.00(ref)			728	91.0	1.00(ref)				
Allele C	217	9.0	66	8.3	0.91(0.68–1.21)	0.495	0.707	72	9.0	1.00(0.75–1.32)	0.972	0.972		
**rs79244935**													2.954	0.228
CC	923	76.9	317	79.3	1.00(ref)			299	74.8	1.00(ref)				
CT	254	21.2	78	19.5	0.89(0.67–1.19)	0.440	0.800	91	22.8	1.11(0.84–1.45)	0.469	0.782		
TT	23	1.9	5	1.3	0.63(0.24–1.68)	0.358	0.796	10	2.5	1.34(0.63–2.85)	0.444	0.987		
CT + TT	277	23.1	83	20.8	0.87(0.66–1.15)	0.333	0.555	101	25.3	1.13(0.87–1.46)	0.377	0.628		
Allelic frequency														
Allele C	2100	87.5	712	89.0	1.00(ref)			689	86.1	1.00(ref)				
Allele T	300	12.5	88	11.0	0.87(0.67–1.11)	0.261	0.653	111	13.9	1.13(0.89–1.43)	0.314	0.628		
***NEIL2***														
**rs804270**													**26**.**842**	**0**.**0001**
GG	368	30.7	113	28.3	1.00(ref)			93	23.3	1.00(ref)				
GC	586	48.8	178	44.5	0.99(0.76–1.30)	0.937	0.986	169	42.3	1.14(0.86–1.52)	0.363	1.037		
CC	246	20.5	109	27.3	**1**.**44(1**.**06–1**.**97)**	**0**.**020**	0.200	138	34.5	**2**.**22(1**.**63–3**.**02)**	**0**.**0001**	**0**.**0001**		
GC + CC	832	69.3	287	71.8	1.12(0.88–1.44)	0.361	0.516	307	76.8	**1**.**46(1**.**12–1**.**90)**	**0**.**005**	**0**.**025**		
Allelic frequency														
Allele G	1322	55.1	404	50.5	1.00(ref)			355	44.4	1.00(ref)				
Allele C	1,078	44.9	396	49.5	**1**.**20(1**.**02–1**.**41)**	**0**.**024**	0.120	445	55.6	**1**.**54(1**.**31–1**.**81)**	**0**.**0001**	**0**.**0001**		
**rs8191613**													2.015	0.365
GG	1,019	84.9	351	87.8	1.00(ref)			346	86.5	1.00(ref)				
GA	176	14.7	47	11.8	0.78(0.55–1.09)	0.147	0.588	53	13.3	0.89(0.64–1.23)	0.476	0.732		
AA	5	0.4	2	0.5	1.16(0.22–6.01)	0.859	1.011	1	0.3	0.59(0.07–5.06)	0.630	0.741		
GA + AA	181	15.1	49	12.3	0.79(0.56–1.10)	0.163	0.815	54	13.5	0.88(0.63–1.22)	0.439	0.627		
Allelic frequency														
Allele G	2214	92.3	749	93.6	1.00(ref)			745	93.1	1.00(ref)				
Allele A	186	7.8	51	6.4	0.81(0.59–1.12)	0.199	0.663	55	6.9	0.88(0.64–1.20)	0.417	0.695		
**rs8191664**													**27**.**630**	**0**.**0001**
GG	1,031	85.9	319	79.8	1.00(ref)			289	72.3	1.00(ref)				
GT	142	11.8	70	17.5	**1**.**59(1**.**17–2**.**18)**	**0**.**003**	0.060	101	25.3	**2**.**54(1**.**91–3**.**38)**	**0**.**0001**	**0**.**0001**		
TT	27	2.3	11	2.8	1.32(0.65–2.68)	0.449	0.748	10	2.5	1.32(0.63–2.76)	0.459	0.918		
GT + TT	169	14.1	81	20.3	**1**.**55(1**.**16–2**.**08)**	**0**.**003**	**0**.**030**	111	27.8	**2**.**34(1**.**78–3**.**08)**	**0**.**000**	**0**.**0001**		
Allelic frequency														
Allele G	2204	91.8	708	88.5	1.00(ref)			679	84.9	1.00(ref)				
Allele T	196	8.2	92	11.5	**1**.**46(1**.**23–1**.**90)**	**0**.**005**	**0**.**050**	121	15.1	**2**.**00(1**.**57–2**.**55)**	**0**.**0001**	**0**.**0001**		

Underlined values show statistical data with significant difference. *All P#-values are adjusted for age, number of sexual partners, age at first intercourse, parities (including full-term pregnancy and abortion at or after 28 weeks) and age at first full-term pregnancy. Pa values were corrected by the method of Benjamin Hochberg (BH method) for multiple testing correction. P* values were analyzed by multinomial regression analysis.

**Table 2:** Association between NEIL1 and NEIL2 genetic variants and the risk of HR-HPV-positive CIN III and CSCCs.

Genotypes	Normal healthy controls N = 191		CIN III N = 310		adjusted OR* (95% CI)	*P*^*#*^	Pa	CSCCs N = 178		adjusted OR* (95% CI)	*P*^*#*^	Pa	*χ*^2^	P*
	N	%	N	%				N	%					
***NEIL1***														
**rs4462560**													0.303	0.860
CC	73	38.2	112	36.1	1.00(ref)			61	34.3	1.00(ref)				
CG	85	44.5	141	45.5	1.08(0.73–1.61)	0.701	1.001	87	48.9	1.23(0.78–1.93)	0.380	1.031		
GG	33	17.3	57	18.4	1.13(0.67–1.89)	0.655	1.008	30	16.9	1.09(0.60–1.98)	0.783	1.063		
AG + GG	118	61.8	198	63.9	1.09(0.75–1.59)	0.638	0.638	117	65.7	1.19(0.78–1.82)	0.431	0.862		
Allelic frequency														
Allele C	231	60.5	365	58.9	1.00(ref)			209	58.7	1.00(ref)				
Allele G	151	39.5	255	41.1	1.07(0.82–1.39)	0.616	1.232	147	41.3	1.08(0.80–1.44)	0.626	1.043		
**rs7182283**													0.604	0.739
GG	52	27.2	91	29.4	1.00(ref)			44	24.7	1.00(ref)				
GT	103	53.9	155	50.0	0.86(0.56–1.31)	0.483	1.073	97	54.5	1.11(0.68–1.81)	0.667	1.152		
TT	36	18.8	64	20.6	1.02(0.60–1.73)	0.954	1.060	37	20.8	1.22(0.66–2.24)	0.532	1.123		
GT + TT	139	72.8	219	70.6	0.90(0.60–1.35)	0.608	0.676	134	75.3	1.14(0.72–1.82)	0.584	0.834		
Allelic frequency														
Allele G	207	54.2	337	54.4	1.00(ref)			185	52.0	1.00(ref)				
Allele T	175	45.8	283	45.6	0.99(0.77–1.28)	0.959	0.959	171	48.0	1.09(0.82–1.46)	0.546	1.092		
**rs7402844**													1.59	0.451
GG	85	44.5	151	48.7	1.00(ref)			77	43.3	1.00(ref)				
GC	66	34.6	104	33.5	0.89(0.59–1.33)	0.564	1.025	66	37.1	1.10(0.70–1.75)	0.674	1.067		
CC	40	20.9	55	17.7	0.77(0.48–1.26)	0.302	1.510	35	19.7	0.97(0.56–1.67)	0.901	1.007		
GC + CC	106	55.5	159	51.3	0.84(0.59–1.21)	0.360	0.900	101	56.7	1.05(0.70–1.59)	0.810	0.900		
Allelic frequency														
Allele G	236	61.8	406	65.5	1.00(ref)			220	61.8	1.00(ref)				
Allele C	146	38.2	214	34.5	0.85(0.65–1.11)	0.235	0.588	136	38.2	1.00(0.74–1.35)	0.996	0.996		
**rs5745920**													0.153	0.926
CC	56	29.3	84	27.1	1.00(ref)			58	32.6	1.00(ref)				
CT	104	54.5	177	57.1	1.14(0.75–1.72)	0.552	1.104	86	48.3	0.80(0.50–1.27)	0.343	1.303		
TT	31	16.2	49	15.8	1.05(0.60–1.85)	0.855	1.006	34	19.1	1.06(0.58–1.95)	0.854	1.082		
CT + TT	135	70.7	226	72.9	1.12(0.75–1.66)	0.590	0.738	120	67.4	0.86(0.55–1.34)	0.498	0.830		
Allelic frequency														
Allele C	216	56.5	345	55.6	1.00(ref)			202	56.7	1.00(ref)				
Allele T	166	43.5	275	44.4	1.04(0.80–1.34)	0.781	1.116	154	43.3	0.99(0.74–1.33)	0.957	1.196		
**rs8030014**													0.062	0.969
AA	55	28.8	99	31.9	1.00(ref)			52	29.2	1.00(ref)				
AG	104	54.5	153	49.4	0.82(0.54–1.24)	0.339	1.356	95	53.4	0.97(0.60–1.55)	0.886	1.052		
GG	32	16.8	58	18.7	1.01(0.59–1.73)	0.980	0.980	31	17.4	1.03(0.55–1.91)	0.939	0.991		
AG + GG	136	71.2	211	68.1	0.86(0.58–1.28)	0.460	0.920	126	70.8	0.98(0.63–1.54)	0.930	0.930		
Allelic frequency														
Allele A	214	56.0	351	56.6	1.00(ref)			199	55.9	1.00(ref)				
Allele G	168	44.0	269	43.4	0.98(0.76–1.26)	0.854	1.068	157	44.1	1.01(0.75–1.34)	0.973	1.081		
**rs11634109**													0.161	0.923
TT	155	81.2	258	83.2	1.00(ref)			147	82.6	1.00(ref)				
TC	34	17.8	47	15.2	0.83 (0.51–1.35)	0.452	1.130	29	16.3	0.90 (0.52–1.55)	0.703	1.027		
CC	2	1.0	5	1.6	1.50 (0.29–7.84)	0.629	1.048	2	1.1	1.05 (0.15–7.58)	0.958	0.958		
TC + CC	36	18.8	52	16.8	0.87 (0.54–1.39)	0.554	0.923	31	17.4	0.91 (0.53–1.54)	0.721	0.901		
Allelic frequency														
Allele T	344	90.1	563	90.8	1.00(ref)			323	90.7	1.00(ref)				
Allele C	38	9.9	57	9.2	0.92 (0.60–1.41)	0.692	1.153	33	9.3	0.93 (0.57–1.51)	0.755	1.079		
**rs79244935**													4.551	0.103
CC	152	79.6	253	81.6	1.00(ref)			131	73.6	1.00(ref)				
CT	36	18.8	48	15.5	0.80(0.50–1.29)	0.362	1.207	39	21.9	1.26(0.76–2.09)	0.379	1.200		
TT	3	1.6	9	2.9	1.80(0.48–6.76)	0.382	1.091	8	4.5	3.09(0.80–11.90)	0.100	0.633		
CT + TT	39	20.4	57	18.4	0.88(0.56–1.38)	0.575	0.821	47	26.4	1.40(0.86–2.27)	0.175	0.583		
Allelic frequency														
Allele C	340	89.0	554	89.4	1.00(ref)			301	84.6	1.00(ref)				
Allele T	42	11.0	66	10.6	0.96(0.64–1.45)	0.862	0.958	55	15.4	1.48(0.96–2.28)	0.075	0.250		
***NEIL2***														
**rs804270**													**9**.**622**	**0**.**008**
GG	55	28.8	74	23.9	1.00(ref)			37	20.8	1.00(ref)				
GC	95	49.7	137	44.2	1.07(0.69–1.66)	0.755	0.944	76	42.7	1.19(0.71–1.99)	0.509	1.209		
CC	41	21.5	99	31.9	**1**.**80(1**.**08–2**.**97)**	**0**.**023**	0.230	65	36.5	**2**.**36(1**.**33–4**.**17)**	**0**.**003**	**0**.**029**		
GC + CC	136	71.2	236	76.1	1.29(0.86–1.94)	0.221	0.737	141	79.2	1.54(0.96–2.49)	0.077	0.385		
Allelic frequency														
Allele G	205	53.7	285	46.0	1.00(ref)			150	42.1	1.00(ref)				
Allele C	177	46.3	335	54.0	**1**.**36(1**.**05–1**.**76)**	**0**.**018**	0.180	206	57.9	**1**.**59(1**.**19–2**.**13)**	**0**.**002**	**0**.**010**		
**rs8191613**													2.898	0.235
GG	159	83.2	273	88.1	1.00(ref)			157	88.2	1.00(ref)				
GA	31	16.2	36	11.6	0.68(0.40–1.14)	0.139	0.927	21	11.8	0.69(0.38–1.25)	0.215	1.021		
AA	1	0.5	1	0.3	0.58(0.04–9.38)	0.703	0.937	0	0.0	—	—			
GA + AA	32	16.8	37	11.9	0.67(0.40–1.12)	0.130	0.650	21	11.8	0.67(0.37–1.20)	0.177	0.443		
Allelic frequency														
Allele G	349	91.4	582	93.9	1.00(ref)			335	94.1	1.00(ref)				
Allele A	33	8.6	38	6.1	0.69(0.43–1.12)	0.134	0.447	21	5.9	0.66(0.38–1.17)	0.156	0.390		
**rs8191664**													**10**.**721**	**0**.**005**
GG	162	84.8	233	75.2	1.00(ref)			122	68.5	1.00(ref)				
GT	24	12.6	70	22.6	**2**.**03(1**.**22–3**.**36)**	**0**.**006**	0.120	51	28.7	**2**.**82(1**.**65–4**.**84)**	**0**.**0001**	**0**.**0001**		
TT	5	2.6	7	2.3	0.97(0.30–3.12)	0.964	1.015	5	2.8	1.33(0.38–4.69)	0.660	1.254		
GT + TT	29	15.2	77	24.8	**1**.**85(1**.**15–2**.**96)**	**0**.**011**	0.110	56	31.5	**2**.**56(1**.**55–4**.**25)**	**0**.**0001**	**0**.**0001**		
Allelic frequency														
Allele G	348	91.1	536	86.5	1.00(ref)			295	82.9	1.00(ref)				
Allele T	34	8.9	84	13.5	**1**.**60(1**.**05–2**.**44)**	**0**.**028**	0.140	61	17.1	**2**.**12(1**.**35–3**.**31)**	**0**.**001**	**0**.**010**		

Underlined values show statistical data with significant difference. *All P#-values are adjusted for age, number of sexual partners, age at first intercourse, parities (including full-term pregnancy and abortion at or after 28 weeks) and age at first full-term pregnancy. Pa values were corrected by the method of Benjamin Hochberg (BH method) for multiple testing correction. P* values were analyzed by multinomial regression analysis.

The frequency of genotype identified that all seven of the *NEIL1* genetic polymorphisms (rs4462560, rs7182283, rs7402844, rs5745920, rs8030014, rs11634109 and rs79244935) and the *NEIL2* rs8191613 genetic polymorphism were not associated with the risk of CIN III and CSCC. The GG, GC, and CC genotype frequencies of *NEIL2* rs804270 were 30.7%, 48.8% and 20.5% in normal healthy controls; 28.3%, 44.5% and 27.3% in CIN III and 23.3%, 42.3% and 34.5% in CSCC, respectively. These results showed that patients with the rs804270 homozygous CC genotype had a significantly higher risk of CIN III (odds ratio[OR] = 1.44; 95% confidence interval[CI]:1.06–1.97) and CSCC (OR = 2.22; 95%CI: 1.63–3.02). We also found that the frequency of C alleles at the rs804270 locus in CIN III (396/800, 49.5%) and CSCC (445/800, 55.6%) were significantly higher than those in normal healthy controls (1078/2400, 44.9%). The OR of the C allele in CIN III was 1.20 (95%CI: 1.02–1.41) and 1.54 (95%CI: 1.31–1.81) in CSCC. Carriers of the C-allele (GC + CC) at rs804270 were associated with a higher risk for CSCC (OR = 1.46; 95%CI: 1.12–1.90).

The GG, GT and TT genotype frequencies of *NEIL2* rs8191664 were 85.9%, 11.8% and 2.3% in the normal healthy controls; 79.8%, 17.5% and 2.8% in CIN III and 72.3%, 25.3% and 2.5% in CSCC, respectively. These results showed that women carrying the heterozygote GT genotype rs8191664 also had a significantly elevated risk of CIN III (OR = 1.59; 95%CI: 1.17–2.18, P = 0.003) and CSCC (OR = 2.54; 95%CI: 1.91–3.38, P = 0.0001). The T allele frequencies of rs8191664 in CIN III (92/800, 11.5%) and CSCC (121/800, 15.1%) were higher than those in normal healthy controls (196/2400, 8.2%). The T allele was associated with a higher risk for both CIN III (OR = 1.46; 95%CI: 1.23–1.90) and CSCC (OR = 2.00; 95%CI: 1.57–2.55), respectively. Carriers of the T-allele (GT + TT) at rs8191664 were associated with a higher risk for CIN III (OR = 1.55; 95%CI: 1.16–2.08) and CSCC (OR = 2.34; 95%CI: 1.78–3.08).

False discovery rate (FDR) multiple testing corrections were applied in order to avoid Type I errors. We found that the frequency of CC or GC + CC genotype of rs804270 and GT or GT + TT genotype of rs8191664 in CSCC group were still higher than normal healthy control group. The specific statistics are shown in the *Pa* value in Table 1.

### The relationship between genetic polymorphisms in NEIL1 and NEIL2 and HR-HPV-positive cases of CIN III and CSCC

In the HR-HPV-positive groups, *NEIL1* rs4462560, rs7182283, rs7402844, rs5745920, rs8030014, rs11634109 and rs79244935, and *NEIL2* rs8191613 genetic polymorphisms were not related to the risk of CIN III or CSCC (Table 2).

However, the homozygous CC genotype of rs804270 showed relatively higher risk for CIN III (OR = 1.80; 95%CI: 1.08–2.97) and CSCC (OR = 2.36; 95%CI: 1.33–4.17). The elevated risk of CIN III and CSCC with the C allele showed an OR of 1.36 (95%CI: 1.05–1.76) and 1.59 (95%CI: 1.19–2.13), respectively. For rs8191664, the heterozygous GT allele also showed a relatively higher risk of CIN III (OR = 2.03; 95%CI: 1.22–3.36) and CSCC (OR = 2.82; 95%CI: 1.65–4.84) in the HR-HPV-positive group. The increased risk of the T allele for CIN III and CSCC showed an OR of 1.60 (95%CI: 1.05–2.44) and 2.12 (95%CI: 1.35–3.31), respectively. Carriers of the T-allele (GT + TT) at rs8191664 were associated with a higher risk for CIN III (OR = 1.85; 95%CI: 1.15–2.96) and CSCC (OR = 2.56; 95%CI: 1.55–4.25).

After FDR multiple testing corrections, we also found that the frequency of CC genotype of rs804270 and GT or GT + TT genotype of rs8191664 in CSCC group were still higher than normal healthy control group. The specific statistics are shown in the *Pa* value in Table 2.

### The association between NEIL2 rs804270 and rs8191664 genetic polymorphisms and sexual and reproductive histories in patients with CIN III and CSCC

Stratified analysis was performed to analyze the association between the *NEIL2* rs804270 and rs8191664 genotypes and age, age at first intercourse, number of sexual partners, number of parities, HR-HPV infection and other clinical data. There was no enrichment between subgroups with CIN III and CSCC and the *NEIL2* rs804270 genetic polymorphism, as show in Table 3. However, as show in Table 4, we observed a higher enrichment of the *NEIL2* rs8191664 genetic polymorphism when patients were subgrouped by the number of sexual partners in CIN III (χ^2^ = 15.577, P = 0.0001) and CSCC (χ^2^ = 26.556, P = 0.0001).

**Table 3:** Association between NEIL2 rs804270 polymorphisms and the risk for CIN III and CSCCs stratified by the sexual, reproductive history.

High risk exposure	Normal healthy controls						χ^2^	*P*	CIN III						χ^2^	*P*	CSCCs						χ^2^	*P*
	GG		GC		CC				GG		GC		CC				GG		GC		CC			
	N	%	N	%	N	%			N	%	N	%	N	%			N	%	N	%	N	%		
Age																								
≤40	182	30.2	299	49.7	121	20.1	0.002	0.965	71	27.5	111	43.0	76	29.5	1.120	0.290	39	24.4	71	44.4	50	31.3	0.957	0.328
>40	186	31.1	287	48.0	125	20.9			42	29.6	67	47.2	33	23.2			54	22.5	98	40.8	88	36.7		
Number of sexual partners																								
≤1	298	30.9	462	48.0	203	21.1	0.057	0.812	85	26.9	144	45.6	87	27.5	0.728	0.394	75	24.3	133	43.0	101	32.7	1.983	0.159
>1	70	29.5	124	52.3	43	18.1			28	33.3	34	40.5	22	26.2			18	19.8	36	39.6	37	40.7		
Age at the first intercourse																								
≤20	118	32.9	179	49.9	62	17.3	2.889	0.089	34	26.2	62	47.7	34	26.2	0.036	0.849	32	25.6	51	40.8	42	33.6	0.311	0.577
>20	250	29.7	407	48.4	184	21.9			79	29.3	116	43.0	75	27.8			61	22.2	118	42.9	96	34.9		
Number of parities																								
≤3	175	31.9	276	50.4	97	17.7	3.107	0.078	40	25.3	73	46.2	45	28.5	0.819	0.366	26	19.8	60	45.8	45	34.4	0.299	0.584
>3	193	29.6	310	47.5	149	22.9			73	30.2	105	43.4	64	26.4			67	24.9	109	40.5	93	34.6		
Age at the first parity																								
≤22	77	32.8	118	50.2	40	17.0	1.724	0.189	27	29.7	44	48.4	20	22.0	0.941	0.332	21	23.6	41	46.1	27	30.3	0.460	0.497
>22	291	30.2	468	48.5	206	21.3			86	27.8	134	43.4	89	28.8			72	23.2	128	41.2	111	35.7		
HR-HPV infection status																								
Positive	55	28.8	95	49.7	41	21.5	2.402	0.121	74	23.9	137	44.2	99	31.9	0.108	0.743	37	20.8	76	42.7	65	36.5	0.032	0.857
Negative	133	31.8	223	53.3	62	14.8			12	25.5	21	44.7	14	29.8			6	26.1	7	30.4	10	43.5		

Stratified analysis were applied by the Kruskale Wallis H. A P value less than 0.05 was considered significant.

**Table 4:** Association between NEIL2 rs8191664 polymorphisms and the risk for CIN III and cervical carcinoma stratified by the sexual, reproductive history.

High risk exposure	Normal healthy controls						χ2	*P*	CIN III						χ2	*P*	CSCCs						χ2	*P*
	GG		GT		TT				GG		GT		TT				GG		GT		TT			
	N	%	N	%	N	%			N	%	N	%	N	%			N	%	N	%	N	%		
Age																								
≤40	519	86.2	70	11.6	13	2.2	0.091	0.763	207	80.2	44	17.1	7	2.7	0.101	0.750	118	73.8	39	24.4	3	1.9	0.352	0.553
>40	512	85.6	72	12.0	14	2.3			112	78.9	26	18.3	4	2.8			171	71.3	62	25.8	7	2.9		
Number of sexual partners																								
≤1	832	86.4	108	11.2	23	2.4	0.812	0.368	265	83.9	44	13.9	7	2.2	**15**.**577**	**0**.**0001**	243	78.6	59	19.1	7	2.3	**26**.**556**	**0**.**0001**
>1	199	84.0	34	14.3	4	1.7			54	64.3	26	31.0	4	4.8			46	50.5	42	46.2	3	3.3		
Age at the first intercourse																								
≤20	305	85.0	42	11.7	12	3.3	0.502	0.479	104	80.0	22	16.9	4	3.1	0.004	0.951	92	73.6	29	23.2	4	3.2	0.113	0.737
>20	726	86.3	100	11.9	15	1.8			215	79.6	48	17.8	7	2.6			197	71.6	72	26.2	6	2.2		
Number of parities																								
≤3	469	85.6	68	12.4	11	2.0	0.070	0.791	124	78.5	29	18.4	5	3.2	0.279	0.598	96	73.3	32	24.4	3	2.3	0.109	0.741
>3	562	86.2	74	11.3	16	2.5			195	80.6	41	16.9	6	2.5			193	71.7	69	25.7	7	2.6		
Age at the first parity																								
≤22	203	86.4	27	11.5	5	2.1	0.054	0.817	74	81.3	15	16.5	2	2.2	0.194	0.660	65	73.0	22	24.7	2	2.2	0.039	0.843
>22	828	85.8	115	11.9	22	2.3			245	79.3	55	17.8	9	2.9			224	72.0	79	25.4	8	2.6		
HR-HPV infection status																								
Positive	162	84.8	24	12.6	5	2.6	0.244	0.621	233	75.2	70	22.6	7	2.3	0.829	0.362	122	68.5	51	28.7	5	2.8	0.211	0.646
Negative	348	83.3	57	13.6	13	3.1			38	80.9	9	19.1	0	0.0			17	73.9	5	21.7	1	4.3		

Underlined values show statistical data with significant difference.

Stratified analysis were applied by the Kruskale Wallis H. A *P* value less than 0.05 was considered significant.

### Association analysis between the NEIL2 rs804270 (G/C) and rs8191664 (G/T) genotypes and the risk of CINIII and CSCC

We analyzed the genotype linkage pattern between the frequencies of both rs804270(G/C) and rs8191664(G/T) genotypes because there was a significant association between these two genetic polymorphisms with the risk of CINIII and CSCC. As shown in Table 5, the GG-TT and CC-TT genotypes were not detected in any of the cases and normal healthy controls. Compared with the reference genotype GG-GG, the CC-GG (OR = 1.42; 95%CI: 1.01–2.00) and CC-GT (OR = 2.07; 95%CI: 1.19–3.61) genotypes were significantly associated with an increased risk of CIN III. A higher risk was detected for GC-GT (OR = 1.91; 95%CI: 1.13–3.23), CC-GG (OR = 1.67; 95%CI: 1.16–2.37) and CC-GT (OR = 6.18; 95%CI: 3.85–9.93) in CSCCs. These data indicated that the genotype linkage pattern of the CC homozygous genotype of rs804270(G/C), and the GT heterozygous genotype of rs8191664(G/T), was associated with an elevated risk for CIN III and CSCC.

**Table 5:** NEIL2 haplotype of rs804270 (G/C) and rs8191664 (G/T) and the risk of all CIN III and CSCCs.

NEIL2 Genotypes^a^	Normal healthy controls		CIN III		adjusted OR^b^ (95% CI)	*P*	CSCCs		adjusted OR^b^ (95% CI)	*P*
	N = 1200		N = 400				N = 400			
	N	%	N	%			N	%		
GG-GG	324	27.0	94	23.5	1.00(ref)		76	19.0	1.00(ref)	
GG-GT	44	3.7	19	4.8	1.49(0.83–2.67)	0.183	17	4.3	1.65(0.89–3.04)	0.111
GG-TT	0	0.0	0	0.0	—	—	0	0.0	—	—
GC-GG	501	41.8	140	35.0	0.96(0.72–1.30)	0.804	133	33.3	1.13(0.83–1.55)	0.441
GC-GT	58	4.8	27	6.8	1.61(0.96–2.68)	0.070	26	6.5	**1**.**91(1**.**13–3**.**23)**	**0**.**016**
GC-TT	27	2.3	11	2.8	1.40(0.67–2.94)	0.367	10	2.5	1.58(0.73–3.40)	0.432
CC-GG	206	17.2	85	21.3	**1**.**42(1**.**01–2**.**00)**	**0**.**043**	80	20.0	**1**.**67(1**.**16–2**.**37)**	**0**.**006**
CC-GT	40	3.3	24	6.0	**2**.**07(1**.**19–3**.**61)**	**0**.**010**	58	14.5	**6**.**18(3**.**85–9**.**93)**	**0**.**0001**
CC-TT	0	0.0	0	0.0	—	—	0	0.0	—	—

Underlined values show statistical data with significant difference. ^a^Genotypes are composed of two polymorphic sites: rs804270(G/C), rs8191664(G/T). ^b^All P-values are adjusted for age, number of sexual partners, age at first intercourse, parities (including full-term pregnancy and abortion at or after 28 weeks) and age at first full-term pregnancy.

In addition, the CC-GG genotype was the most common genotype linkage pattern in the CIN III [85/(85 + 24), 77.98%], CSCC [80/(80 + 58), 57.97%] and nornal healthy control groups [206/(206 + 40), 83.74%] which carried the CC genotype at rs804270(G/C). Similarly, the GC-GT genotype was the most common genotype linkage pattern in the nornal healthy control [58/(44 + 58 + 40), 40.85%] and the CIN III [27/(19 + 27 + 24), 38.57%] groups, which carried the GT genotype at rs8191664 (G/T). However, the CC-GT genotype was the most common genotype linkage pattern in the CSCC group [58/(17 + 26 + 58), 57.43%] which carried the GT genotype at rs8191664 (G/T). These results indicate that there was a specific genotype linkage pattern between rs804270(CC) and rs8191664(GT). In other words, these specific genotype linkage patterns were associated with a higher risk of CIN III or CSCC. The genotypes of GC-GT, CC-GG, and CC-GT of rs804270 and rs8191664 SNP in the *NEIL2* gene may act as a genetic predictive biomarker of susceptibility for CIN III and CSCC.

### The linkage disequilibrium and haplotype analysis of three SNP loci in NEIL2 gene

Because the genotypes of two SNP loci in *NEIL*2 were significantly correlated with the susceptibility of CIN III and CSCC, we further analyzed the linkage disequilibrium and haplotype of all three SNP loci in *NEIL*2 with the SHEsis software. The pairing analysis showed that the D’ and r^2^ values did not have statistical significance, there was no linkage disequilibrium between the three SNPs each other, this also meant that there is no specific haplotype between the three SNP. However, we noted that there may be a trend of linkage disequilibrium between rs8191613 and rs8191664 in CIN III group(D’ = 0.768), while in CSSS group, there may be a trend of linkage disequilibrium between rs804270and rs8191664(D’ = 0.344). The specific statistical results are shown in Tables 6 and 7.

**Table 6:** D’ value of the linkage disequilibrium analysis between SNPs of NEIL2 gene.

Pair-loci	Normal healthy Control	CIN III	CSCC
rs804270-rs8191613	0.025	0.016	0.028
rs804270-rs8191664	0.108	0.095	0.344
rs8191613-rs8191664	0.435	0.768	0.099

**Table 7:** r^2^ value of the linkage disequilibrium analysis between SNPs of NEIL2 gene.

Pair-loci	Normal healthy Control	CIN III	CSCC
rs804270-rs8191613	0.000	0.000	0.000
rs804270-rs8191664	0.001	0.001	0.017
rs8191613-rs8191664	0.001	0.005	0.004

### The mRNA and protein expression levels of NEIL2 in CSCC tissues with different rs804270 (G/C) or rs8191664 (G/T) genotypes

**Figure 1:**
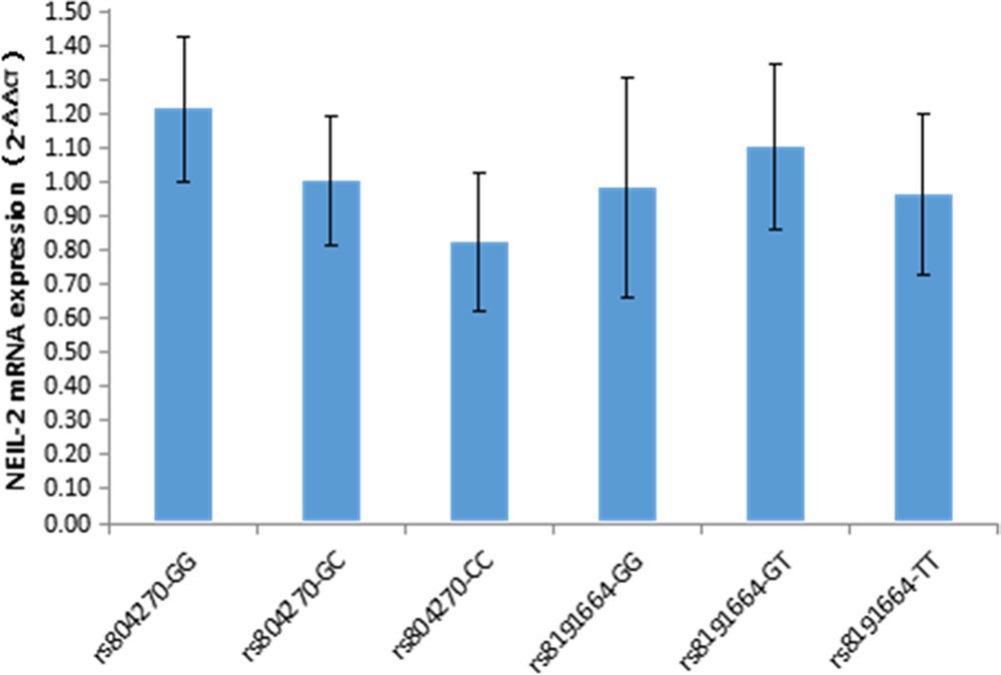
mRNA expression of NEIL2 in CSCCs with different genetic polymorphisms. rs804270-GG: rs804270 genotype is GG; rs804270-GC: rs804270 genotype is GC; rs804270-CC: rs804270 genotype is CC; rs8191664-GG: rs8191664 genotype is GG; rs8191664-GT: rs8191664 genotype is GT; rs8191664-TT: rs8191664 genotype is TT. The rs804270-GG and rs8191664-GG genotypes were used as the control groups of mRNA expression in different genotypes of rs804270 and rs8191664, respectively.

**Figure 2:**
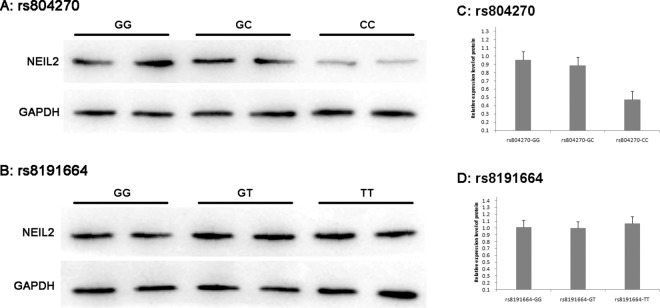
Protein expression of NEIL2 in CSCCs with different genetic polymorphisms. (**A**,**B)** Immunoblot, the molecular weight of NEIL2 and GAPDH protein is 37 kDa and 36 kDa respectively; (**C**,**D**) Analysis of protein relative expression of different genotypes. In rs804270 SNP, GC compared with GG, t = 1.819, P = 0.074; CC compared with GG, t = 16.789, P = 0.000; Compared with GC, t = 12.909, P = 0.000. In rs8191664 SNP, GT compared with GG, t = 0.437, P = 0.663; T T compared with GG, t = −0.539, P = 0.592; T T compared with GT, t = −0.511, P = 0.614.

The number of cases and the frequencies of the GG, GC, and CC genotypes of rs804270 among the 92 CSCC patients were 22 (23.9%), 38 (41.3%), and 32 (34.8%) cases, respectively. When the rs804270(GG) group was used as a control group, the expression of *NEIL2* mRNA in patients with rs804270(CC) (0.824 ± 0.201) was significantly lower(30% reduction, P < 0.001) than that in patients with rs804270(GG) (1.215 ± 0.213) and rs804270(GC) (1.003 ± 0.188) (Fig. 1). Similarly, in the rs804270(CC) group, the protein expression of *NEIL2* also was significantly lower (50% reduction, P < 0.001) (Fig. 2A,C).

The number of cases and the frequencies of the GG, GT and TT genotypes of rs8191664 among the 92 CSCC patients, were 63 (68.5%), 26 (28.3%), and 3 (3.2%) cases, respectively. When the rs8191664(GG) group was used as a control group, there was no significant difference in the expression of *NEIL2* mRNA among patients with rs8191664(GG) (0.985 ± 0.321), rs8191664(GT) (1.103 ± 0.244) and rs8191664(TT) (0.964 ± 0.235) (Fig. 1). Similarly, there was no significant difference in the expression of *NEIL2* protein when compared among different genotype groups (Fig. 2B,D).

## Discussion

Aerobic respiration can produce ROS via a range of pathological processes^18,19^. These chemicals or free radicals can cause DNA damage^20^, which lead to genomic instability and eventually lead to the initiation and development of malignant tumors^21–24^. Most of the damaged bases are removed and repaired by DNA glycosylase and the base excision repair system (BER)^25–28^. *NEIL1* and *NEIL2* are key functional proteins in the BER pathway.

The *NEIL1* gene participates in the first step of the BER repair mechanism^29^. It was reported that FapyA or 5S-6R thymidine glycol cannot be excised by neutral trehalase 1 (NTH1) or 8-oxoguanine glycosylase (OGG1), but can be repaired by *NEIL1*. However, embryonic stem cells lacking *NEIL1* expression were approximately twice as sensitive to low-level radiation-induced damage as normal cells^30^. Studies have also shown that *NEIL1* protein is more efficient than 8-oxoG in the removal of thymidine glycol and 5-hydroxyuracil from damaged DNA^31,32^. However, *NEIL1*-knockout mice developed metabolic disorder syndrome, characterized by severe obesity, dyslipidemia and fatty liver^33^.

Three *NEIL1* promoter genetic polymorphisms (c.-3769C > T, c.-3170T > G and c.-2681TA) were found to play an important role in the development of gastric cancer^15^. Zhai *et al*. found that the *NEIL2* rs804270(CC) allele was associated with the advanced stage of oropharyngeal and oral squamous cell carcinoma. However, these authors did not find any risk associated with the *NEIL1* rs4462560 and rs7182283 genetic polymorphisms^14^. In present study, we chose seven SNPs with a MAF value of more than 5% in the *NEIL1* gene and found that none of these SNPs were associated with susceptibility to CSCC or its precancerous lesion CIN III. Our results show that genetic polymorphisms in the introns of *NEIL1* were not related to the occurrence of cervical carcinoma. However, further studies are now required to investigate the relationship between genetic polymorphisms in the promoter region of *NEIL1* and the risk of cervical carcinoma. It is possible that genetic polymorphisms in the promoter region may alter the protein expression of the *NEIL1* gene, thereby altering cell behavior. However, because the three SNPs in the *NEIL1* promotor region have small MAF in the general population, it is necessary to carry out additional studies featuring a larger sample size to study this association more robustly.

*NEIL2* exhibits the strongest activity for 5-hydroxyuracil and weakest activity for 5-hydroxycytosine, 8-oxoG, thymine glycol and dihydrouracil^34^. Low expression levels of *NEIL2* may cause somatic cell DNA mutation and copy number variation, thus leading to genomic instability, oncogene activation and inhibition the expression of tumor suppressor genes^35,36^. Elingarami *et al*. evaluated the potential association between *NEIL2* SNPs (rs804270, 5′-UTR promoter region) and susceptibility to gastric carcinoma, and assessed whether genotypes affected the expression of *NEIL2* mRNA^37^, they reported that there is an increased risk of gastric cancer in patients with genetic variants of *NEIL2* SNP(rs804270). Moreover, studies showed that the expression of *NEIL2* mRNA was significantly different when compared across different *NEIL2* genotypes. In present study, we found that the frequencies of the GG, GC, and CC genotypes of *NEIL2* rs804270 were 30.7%, 48.8% and 20.5% in the normal healthy controls, 28.3%, 44.5% and 27.3% in CIN III and 23.3%, 42.3% and 34.5% in CSCC, respectively. Furthermore, there was a significant correlation between the CC homozygote of rs804270 and the risk of CIN III and CSCC. Carriers of the C-allele (GC + CC) at rs804270 were associated with a higher risk for CSCC. Considering that *NEIL2* rs804270 is located in the 5′-UTR promotor region, we considered that genetic variation might affect the expression of the *NEIL2* gene; we therefore measured the NEIL2 expression of the mRNA and protein. Finally, we concluded that the mRNA and protein expression of *NEIL2* in pathological tissues with the genotype CC of *NEIL2* SNP (rs804270) were significantly reduced. These results indicated that the effect of the *NEIL2* SNP (rs804270) on the susceptibility to cervical carcinoma may be caused by alterating the expression of *NEIL2*, and resulting in a subsequent decline in repair to the damaged genome, thus causing genomic instability and tumor initiation.

In this study, we also evaluated the association between genetic polymorphisms in the exonic regions of *NEIL2* and the risk of CSCC. The heterozygous GT genotype of *NEIL2* rs8191664 was associated with an elevated risk of both CIN III and CSCC. Carriers of the T-allele (GT + TT) at rs8191664 showed a higher risk for CIN III and CSCC. Interestingly, although the GT heterozygous genotype at the rs8191664 locus was identified as a high risk factor, the TT homozygous genotype was not susceptible to disease. This may be due to the fact that there was a low incidence of the TT homozygous genotype in the population. Only 2.3%, 2.8% and 2.5% of the normal healthy control, CIN III and CSCC were identified in present study, thus resulted in fluctuations in the statistical significance.

We also found that the mRNA and protein expression of *NEIL2* did not differ significantly between any genotypes of *NEIL2* rs8191664. We postulate that the *NEIL2* rs8191664 (R257L) SNP does not change *NEIL2* expression, but instead, results in a non-synonymous change in amino acid sequence. This may result in the change of the spatial structure of protein functional domains, thus affecting functional activity. Dy *et al*. found that compared with wild-type cells, the level of endogenous DNA damage in cells featuring the *NEIL2* variant rs8191664 (G/T; R257L) was increased^38^. The reduced levels of DNA repair activity in cells featuring the *NEIL2* rs8191664 (R257L) missense mutation can induce genomic instability that ultimately leads to the initiation of cervical carcinoma.

In present study, as shown in Tables 3 and 4, we further stratified the clinical data relating to patient age, age at first sexual intercourse, the number of parities and age at first parity. We found that there were no associations between these features and either of the two *NEIL2* SNPs [rs804270 and rs8191664 (R257L)]. These results also indicated that there was no correlation between the two *NEIL2* SNPs [rs804270 and rs8191664 (R257L)] and HR-HPV infection. However, there was a higher enrichment of the *NEIL2* rs8191664 GT or TT genotypes in CIN III and CSCC when there was more than one sexual partner. In a family and twin studies, Sanders AR *et al*. found a significant association between different sexual orientations and SNPs on chromosomes 8, 13, 14 and X^39^. Furtherly, Pearce E *et al*. found that SNP in oxytocin and dopamine receptor gene was closely related to a person’s sexual attitudes and behavior, which confirmed the relationship between social behavior with the neurochemical differences caused by SNP in human gene^40^. This provides a theoretical basis for understanding the correlation between SNP and behavior at the molecular biological level. Because the relationship between behavior and gene is more complex than that between tumor and gene, it is related to more gene information. The study of the relationship between phenotype and gene involves more genes or loci. In order to better identify this correlation, we believe that not only the sample size of the study needs to be increased, but also the related polymorphism sites need to be increased. We’d better do further research on genome-wide association and gene function studies.

We compared the *NEIL2* rs804270 (G/C) and rs8191664 (G/T) genotypes with the reference genotype GG-GG and found that the CC-GG and CC-GT genotypes were significantly associated with an increased risk of CIN III. For CSCC, the risk was much greater for the GC-GT, CC-GG and CC-GT genotypes. In particular, the CC-GT genotype has a greater impact on disease susceptibility than when these two loci were analyzed separately, the OR values for CINIII and CSCC were 2.07 and 6.18, respectively. A higher OR suggested a synergistic effect between these two genetic polymorphisms in the *NEIL2* gene. It is possible that this synergistic effect promoted the development of CIN III to eventually lead to cervical carcinoma. We also observed that neither the GG genotype nor G allele conferred the risk of disease when rs8191664 was analyzed separately, although the CC-GG genotype was still at risk. This may be because the CC genotype at rs804270 had a greater impact on disease susceptibility, while rs8191664 was not a protective factor. The effect of the CC genotype at the rs804270 locus could not be eliminated by rs8191664 GG genotype. At the same time, we further analyzed the linkage disequilibrium and haplotype of three SNP loci in *NEIL2* gene. There was no linkage disequilibrium among the three SNPs each other. However, we noted that there may be a trend of linkage disequilibrium between rs8191613 and rs8191664 in CIN III group, while in CSSS group, there may be a trend of linkage disequilibrium between rs804270 and rs8191664.

In summary, these results suggested that two genetic polymorphisms (rs804270 and rs8191664) in the *NEIL2* gene were associated with susceptibility to CIN III and CSCC. This effect is likely to be due to alterations in *NEIL2* repair activity arising from a change in protein expression or functional domain structure. The GC-GT, CC-GG and CC-GT genotypes at rs804270, and rs8191664 SNPs in the *NEIL2* gene, may act as a genetic biomarker to predict the susceptibility to CIN III and CSCC.

## Methods

### Subject selection and sexual, reproductive, and HR-HPV infection history characteristics

Four hundred CSCCs, four hundred CIN III and one thousand and two hundred normal healthy controls were selected for this study from Chinese population. Their pathological diagnosis was confirmed by two gynecologic pathologists. Normal, healthy female volunteers served as controls and were recruited during gynecological examinations from 2004 to 2008. Normal healthy controls were selected according to the criteria of no pathological cytology findings, endometriosis, gynecological neoplasm, and other solid tumors or immune diseases. Of these, 201 CSCC patients, 357 CIN III patients and 609 normal healthy controls agreed to obtain cervical brushing exfoliated cells to do HR-HPV detection.The infection rates of HR-HPV in CSCC, CIN III and normal healthy controls group were 88.6%, 86.8% and 31.4% respectively. The infection rate of HR-HPV in patients with CIN III and CSCC was significantly higher than that in healthy controls (P < 0.001, χ^2^ = 277.1; P < 0.001, χ^2^ = 199.3, respectively).

In normal healthy control group, CIN III group and CSCC group, the number of patients younger than or older than 40 years old was 602/598, 258/142 and 160/240, respectively. Compared with the normal healthy control group, the age of CSCC group was significantly higher than that of 40 years old (P < 0.001, χ^2^ = 12.4), while the age of CIN III group was lower than that of 40 years old (P < 0.001, χ^2^ = 24.7). In CIN III and CSCC groups, more individuals with more than three parities were found(P = 0.031, χ^2^ = 4.6; P < 0.001, χ^2^ = 20.5, respectively). In CSCC, CIN III and the normal healthy control group, stratified analysis by age at the time of first sexual intercourse (patients were grouped under 20 years old or over), number of sexual partners (patients were grouped by one or more partners) and age at the time of first birth (patients were grouped under 20 years old) showed that there was no statistical difference in this stratification within the group.

### Ethical statement

This study was approved by the Medical Ethics Committee of Women’s Hospital Affiliated to Medical School of Zhejiang University (No. 2004002). Informed consent was signed by both patients and normal controls. All the research methods protocols were followed under the approved guidelines and regulations.

### SNP selection

We searched for SNPs in the *NEIL1* and *NEIL2* genes from SNP Library Established by National Library of Medicine (website: www.ncbi.nlm.nih.gov). By utilizing filters (SNP, minor allele frequency (MAF) from 0.05 to 0.5), we obtained seven effective SNPs in the *NEIL1* gene. Interestingly, these seven SNPs were located in introns. By utilizing filters for the *NEIL2* gene (SNP, missense, MAF from 0.05 to 0.5), we only obtained three effective SNPs in the *NEIL2* gene.

The ten SNPs are listed as follows: rs4462560 (C/G), rs7182283 (G/T), rs7402844 (C/G), rs5745920 (C/T), rs8030014 (A/G), rs11634109 (C/T), and rs79244935 (C/T) in the *NEIL1* intronic region; rs804270 (C/G) in the *NEIL2* 5′ UTR region, rs8191613 (A/G) in the *NEIL2* intronic region, and rs8191664 (G/T) in the *NEIL2* exonic region.

### gDNA extraction and SNP genotyping

According to the manufacturer’s protocol, we use the whole genome DNA(gDNA) extraction kit to extract genomic DNA from anticoagulant peripheral blood. (Sangon Bio Co., Shanghai, China). Genomic DNA dissolves in deionized water and is cryopreserved.

Ten SNP genotypes in *NEIL1* and *NEIL2* genes were determined by modified allele mismatch amplification polymerase chain reaction (MAMA-PCR), as described earlier^41^. Specific forward and reverse primers and product lengths for MAMA-PCR are shown in Table S1.

Briefly, the PCR reaction was carried out in a total 20 µL volume reaction mixture containing 20 ng gDNA, 5.0 pmol forward and reverse primer, 0.25 mm dNTP and 1.0U Taq DNA polymerase (TAKARA Co., Dalian, China).The conditions of PCR reaction were as follows: initial denaturation at 94 °C for 5 minutes, followed by 35 cycles: denaturation at 94 °C for 30 seconds, annealing at 55–58 °C for 30 seconds (different primer pairs required different annealing temperatures), and elongation at 72 °C for 30 seconds. At last, a final elongation at 72 °C was performed for 10 minutes. PCR products were analyzed by 2% agarose gel electrophoresis followed by ethidium bromide staining. All the results were measured twice by two technicians with double blind method, and the repeatability of the experiment was completely consistent. In order to further verify the reliability of MAMA-PCR, we selected 5 samples of three genotypes of each locus for using DNA sequencing. In our study, there are 10 loci in total, so the total number of sequencing is: 10 loci * 3 genotypes * 5 samples = 150. The sequencing results of these 150 cases are identical with those of MAMA-PCR. The electropherogram was shown in Fig. S1.

### Detection of HR-HPV infection

Hybrid Capture II kit(Digene Diagnostics Co., USA) with probe B was used to detect HR-HPV infection. Probe B can detect 13 subtypes of HR-HPV in total (including 16, 18, 31, 33, 35, 39, 45, 51, 52, 56, 58, 59, and 68). The cervical exfoliated cells for testing were obtained using Digene cervical sampler according to the manufacturer’s instructions.

### Detection of NEIL2 mRNA expression

Ninety-two freshly-frozen CSCC tissue samples were used for RNA isolation and *NEIL2* gene expression analysis. According to the manufacturer’s procedure, TRIzol reagent(Invitrogen Co., USA) was used to extract total RNA from tissues. The total RNA of each sample was digested by RNase-free DNase I. The purity and quantity of RNA was confirmed with a NanoDrop 2000 (Thermo Fisher). Absorbance at 260/280 of total RNA was between 1.8 and 2.0. The synthesized cDNA serves as a template for qRT-PCR to detect mRNA expression. The reaction conditions of qRT-PCR were 95 °C 30 seconds, followed by 40 cycles: 95 °C, 5 seconds; 60 °C, 35 seconds. The primer sequences for detecting *NEIL2* mRNA (NM_001135746.2) were 5′-ATGGAAAGAAATTATTCCTT-3′; and 5′-CAGAATCATCCTCGCCCTGG-3′. *GAPDH* was served as an internal reference for qRT-PCR. The primer sequences of *GAPDH* mRNA were 5′-GAGAAGGCTGGGGCTCATTT-3′ and 5′-AGTGATGGCATGGACTGTGG-3′. The length of PCR products of *GAPDH* and *NEIL2* were 231 bp and 204 bp, respectively. All the PCR reactions were performed on ABI’s VIIA 7 DX system. The ΔCt for *NEIL2* mRNA expression was calculated compared with the Ct of internal reference *GAPDH*. The mRNA expression of *NEIL2* was calculated by formula: 2^−ΔΔCt^.

### Immunoblotting for NEIL2 protein

*NEIL2* protein expression was detected in 92 CSCC tissue samples by immunoblotting. Simply, the tissue sample was minced on ice, dissolved in RIPA tissue lysate buffer, and then homogenized. The supernatant was collected and the protein concentration was detected after rotating the test tube at 4 °C for 1 hour and centrifuging at 12,000 rpm at 4 °C.

Protein lysate (10 μL) was electrophoretic separated on 8% polyacrylamide gel, and then the imprinted proteins were transferred to 0.45 µm PVDF membranes. The PVDF membrane was cultured overnight with the primary antibodies *NEIL1* (1:2000) (Proteintech Co., USA) *NEIL2* (1:1000) (Invitrogen Co., USA), and *GAPDH* (1:5000) (Proteintech Co., USA) at 4 °C after 1 hour blocking with 5% nonfat-milk. The membrane was washed three times with TBS buffer containing 0.05% Tween-20, and then incubated for 1 hour with an HRP-conjugated secondary antibody. After fluorescent labeling with ECL substrate, Image Quant LAS 4000 mini (GE Healthcare Co., USA) was used to image the ECL membranes, and then quantitative analysis of the proteins was performed.

### Statistical analysis

In order to analyze the correlation between genotype and the risk of CSCC, binary logistic regression analysis was used to obtain odds ratio (OR), 95% confidence interval (CI) and P value. The normal control group was acted as a reference. FDR adjusted p values were corrected by the method of Benjamin Hochberg (BH method) for multiple testing correction. Kruskal-Wallis H test was used for stratified analysis of reproductive and sexual history and genotype distribution frequency. Multinomial regression analysis was performed among the different groups for different genotypes, and less than 0.05 of P value indicates the model fitting is significant. To analyze the differences in the expression of mRNA or protein, ANOVA (Fisher’s Least Difference test) or Student’s two-tailed t-test was used for statistical analysis. Statistical significance level was set at P ≤ 0.05, and it was a bilateral test. All statistical processes are completed by SPSS software (Version 18.0 for Windows). The linkage disequilibrium of three SNP loci in *NEIL2* gene was analyzed with the SHEsis software^42^.

## Supplementary information

Supplementary information 1

Supplementary information 2

Supplementary information 3
