# Supplementary material for: Cervical carcinoma risk associate with genetic polymorphisms of NEIL2 gene in Chinese population and its significance as predictive biomarker
Source: Sci Rep. 2020 Mar 20;10:5136. doi: 10.1038/s41598-020-62040-9 (PMC7083954; doi:10.1038/s41598-020-62040-9)
Supplement: Supplementary file 1 — Supplementary information 1 [file 41598_2020_62040_MOESM1_ESM.doc]

**Title:** Cervical carcinoma risk associate with genetic polymorphisms of NEIL2 gene in Chinese population and its significance as predictive biomarker

**Authors:** Feng Ye ^1,2^, Jia Liu^3^, Hanzhi Wang^1,2^, Xiaojing Chen^1,2^,Qi Cheng^1,2^, Huaizeng Chen ^1,2^

**Authors:** Feng Ye ^1,2^, Jia Liu^3^, Hanzhi Wang^1,2^, Xiaojing Chen^1,2^,Qi Cheng^1,2^, Huaizeng Chen ^1,2^

**Affiliations:**

1 Central Laboratory of Women’s Hospital, School of Medicine, Zhejiang University, Hangzhou City, Zhejiang Province, China;

2 Women’s Reproductive Health Key Laboratory of Zhejiang Province, Women’s Hospital, School of Medicine, Zhejiang University, Hangzhou City, Zhejiang Province, China;

3 Department of Gynecology, Women’s Hospital, School of Medicine, Zhejiang University, Hangzhou City, Zhejiang Province, China

**Corresponding Authors:** Huaizeng Chen, MD. ([**chenhz@zju.edu.cn**](mailto:chenhz@zju.edu.cn)); Central Laboratory of Women’s Hospital, School of Medicine, Zhejiang University, Hangzhou City, Zhejiang Province, China;Tel: 0086-571-87061878


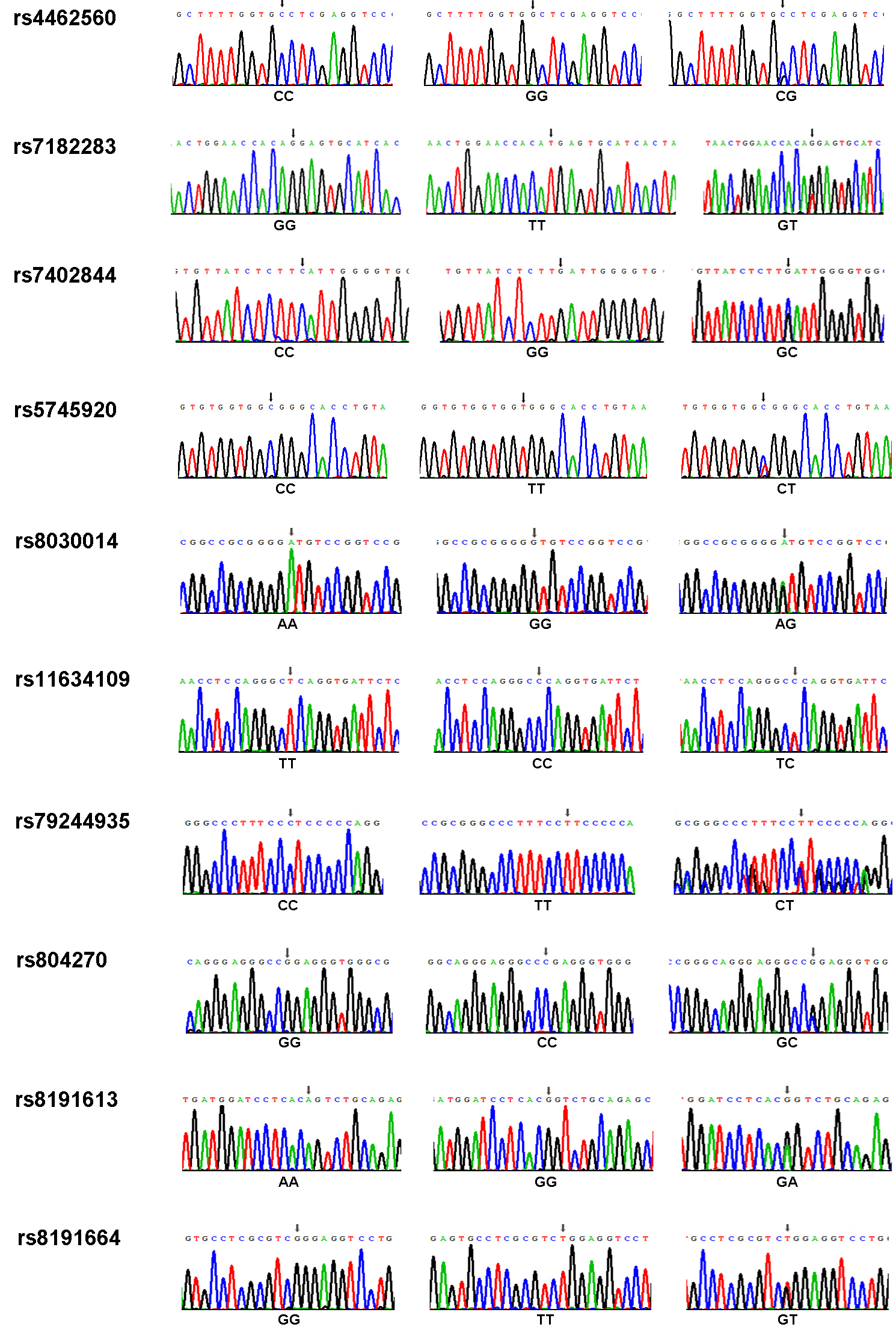


**Figure S1.** Electropherogram of SNPs in NEIL1 and NEIL2 genes
