# Supplementary material for: Cervical carcinoma risk associate with genetic polymorphisms of NEIL2 gene in Chinese population and its significance as predictive biomarker
Source: Sci Rep. 2020 Mar 20;10:5136. doi: 10.1038/s41598-020-62040-9 (PMC7083954; doi:10.1038/s41598-020-62040-9)
Supplement: Supplementary file 2 — Supplementary information 2 [file 41598_2020_62040_MOESM2_ESM.doc]

**Title:** Cervical carcinoma risk associate with genetic polymorphisms of NEIL2 gene in Chinese population and its significance as predictive biomarker

**Authors:** Feng Ye ^1,2^, Jia Liu^3^, Hanzhi Wang^1,2^, Xiaojing Chen^1,2^,Qi Cheng^1,2^, Huaizeng Chen ^1,2^

**Affiliations:**

1 Central Laboratory of Women’s Hospital, School of Medicine, Zhejiang University, Hangzhou City, Zhejiang Province, China;

2 Women’s Reproductive Health Key Laboratory of Zhejiang Province, Women’s Hospital, School of Medicine, Zhejiang University, Hangzhou City, Zhejiang Province, China;

3 Department of Gynecology, Women’s Hospital, School of Medicine, Zhejiang University, Hangzhou City, Zhejiang Province, China

**Corresponding Authors:** Huaizeng Chen, MD. ([**chenhz@zju.edu.cn**](mailto:chenhz@zju.edu.cn)); Central Laboratory of Women’s Hospital, School of Medicine, Zhejiang University, Hangzhou City, Zhejiang Province, China;Tel: 0086-571-87061878

**Supplementary Tables:**

**Table S1:** The specific forward, reverse primers and PCR product length for detecting the SNPs of NEIL1 and NEIL2 gene

| **Gene Name** | **SNP No.** | **Forward primer** | | **Reverse primer** | **Product length** |
| --- | --- | --- | --- | --- | --- |
| **NEIL1** | rs4462560**[C/G]** | For “**C**” | 5’-TCTTCACTGGCTTTTGGTCC-3’ | 5’-TCCCCAGCCTAAAGCAGGG-3’ | 246bp |
|  |  | For “**G**” | 5’-TCTTCACTGGCTTTTGGTCG-3’ |  |  |
|  | rs7182283**[G/T]** | For “**G**” | 5’-CTGATTAACTGGAACCACATG-3’ | 5’-ATTGTGAAAGGTGAAAAATA-3’ | 210bp |
|  |  | For “**T**” | 5’-CTGATTAACTGGAACCACATT-3’ |  |  |
|  | rs7402844**[C/G]** | For “**C**” | 5’-CAATGTGTGTTATCTCTAC-3’ | 5’-GCATCATATCAGGAGGCACC-3’ | 278bp |
|  |  | For “**G**” | 5’-CAATGTGTGTTATCTCTAG-3’ |  |  |
|  | rs5745920**[C/T]** | For “**C**” | 5’-AATTAGCCAGGTGTGGTGGCC-3’ | 5’-AATATATAAAAGAATCCTTG-3’ | 232bp |
|  |  | For “**T**” | 5’-AATTAGCCAGGTGTGGTGGCT-3’ |  |  |
|  | rs8030014**[A/G]** | For “**A**” | 5’-CCCGGGGGCGGCCGCGGGCA-3’ | 5’-GCTGTCCCTCTGTCCCAGAC-3’ | 315bp |
|  |  | For “**G**” | 5’-CCCGGGGGCGGCCGCGGGCG-3’ |  |  |
|  | rs11634109**[C/T]** | For “**C**” | 5’-CAGCCTTAACCTCCAGGGGC-3’ | 5’-GGTGTGGTGGCTCACACCTG-3’ | 212bp |
|  |  | For “**T**” | 5’-CAGCCTTAACCTCCAGGGGT-3’ |  |  |
|  | rs79244935**[C/T]** | For “**C**” | 5’- GCTGCCGCGGGCCCTTTCGC-3’ | 5’-CCTGGCGCGGGGGAGCGGCG-3’ | 247bp |
|  |  | For “**T**” | 5’- GCTGCCGCGGGCCCTTTCGT-3’ |  |  |
| **NEIL2** | rs804270**[C/G]** | For “**C**” | 5’-CCCCCGGGCAGGGAGGGCGC-3’ | 5’-GACAAAACCGCCAGGCTGGA-3’ | 317bp |
|  |  | For “**G**” | 5’-CCCCCGGGCAGGGAGGGCGG-3’ |  |  |
|  |  | For “**T**” | 5’-CCCCCGGGCAGGGAGGGCGT-3’ |  |  |
|  | rs8191613**[A/G]** | For “**A**” | 5’-ACCCTTGATGGATCCTCACGA-3’ | 5’-ATCAGAGCCCACGAAAATCA-3’ | 240bp |
|  |  | For “**G**” | 5’-ACCCTTGATGGATCCTCACGG-3’ |  |  |
|  |  | For “**T**” | 5’-ACCCTTGATGGATCCTCACGT-3’ |  |  |
|  | rs8191664**[G/T]** | For “**A**” | 5’-GTCCTGAGTGCCTCGCGTGA-3’ | 5’-TCTGGCTCCTCTGACAACTG-3’ | 230bp |
|  |  | For “**T**” | 5’-GTCCTGAGTGCCTCGCGTGT-3’ |  |  |
|  |  | For “**G**” | 5’-GTCCTGAGTGCCTCGCGTGG-3’ |  |  |
|  |  | For “**C**” | 5’-GTCCTGAGTGCCTCGCGTGC-3’ |  |  |
