# Supplementary material for: Cervical carcinoma risk associate with genetic polymorphisms of NEIL2 gene in Chinese population and its significance as predictive biomarker
Source: Sci Rep. 2020 Mar 20;10:5136. doi: 10.1038/s41598-020-62040-9 (PMC7083954; doi:10.1038/s41598-020-62040-9)
Supplement: Supplementary file 3 — Supplementary information 3 [file 41598_2020_62040_MOESM3_ESM.doc]

**Title:** Cervical carcinoma risk associate with genetic polymorphisms of NEIL2 gene in Chinese population and its significance as predictive biomarker

**Authors:** Feng Ye ^1,2^, Jia Liu^3^, Hanzhi Wang^1,2^, Xiaojing Chen^1,2^,Qi Cheng^1,2^, Huaizeng Chen ^1,2^

**Affiliations:**

1 Central Laboratory of Women’s Hospital, School of Medicine, Zhejiang University, Hangzhou City, Zhejiang Province, China;

2 Women’s Reproductive Health Key Laboratory of Zhejiang Province, Women’s Hospital, School of Medicine, Zhejiang University, Hangzhou City, Zhejiang Province, China;

3 Department of Gynecology, Women’s Hospital, School of Medicine, Zhejiang University, Hangzhou City, Zhejiang Province, China

**Corresponding Authors:** Huaizeng Chen, MD. ([**chenhz@zju.edu.cn**](mailto:chenhz@zju.edu.cn)); Central Laboratory of Women’s Hospital, School of Medicine, Zhejiang University, Hangzhou City, Zhejiang Province, China;Tel: 0086-571-87061878

**Table S2:** Hardy-Weinberg Equilibrium test for the alleles of SNPs of NEIL1 and NEIL2 genes in normal healthy controls

| Genotypes | | | Control (N=1200) | |
| --- | --- | --- | --- | --- |
|  |  |  | N | P |
| ***NEIL-1*** | | |  |  |
|  | **rs4462560** | |  | 0.042 |
|  |  | CC | 433 |  |
|  |  | CG | 547 |  |
|  |  | GG | 220 |  |
|  | **rs7182283** | |  | 0.041 |
|  |  | GG | 356 |  |
|  |  | GT | 627 |  |
|  |  | TT | 217 |  |
|  | **rs7402844** | |  | **0.0001** |
|  |  | GG | 527 |  |
|  |  | GC | 402 |  |
|  |  | CC | 271 |  |
|  | **rs5745920** | |  | **0.0001** |
|  |  | CC | 374 |  |
|  |  | CT | 664 |  |
|  |  | TT | 162 |  |
|  | **rs8030014** | |  | **0.0001** |
|  |  | AA | 323 |  |
|  |  | AG | 664 |  |
|  |  | GG | 213 |  |
|  | **rs11634109** | |  | 0.947 |
|  |  | TT | 993 |  |
|  |  | TC | 197 |  |
|  |  | CC | 10 |  |
|  | **rs79244935** | |  | 0.262 |
|  |  | CC | 923 |  |
|  |  | CT | 254 |  |
|  |  | TT | 23 |  |
| ***NEIL-2*** | | |  |  |
|  | **rs804270** | |  | 0.649 |
|  |  | GG | 368 |  |
|  |  | GC | 586 |  |
|  |  | CC | 246 |  |
|  | **rs8191613** | |  | 0.373 |
|  |  | GG | 1,019 |  |
|  |  | GA | 176 |  |
|  |  | AA | 5 |  |
|  | **rs8191664** | |  | **0.0001** |
|  |  | GG | 1,031 |  |
|  |  | GT | 142 |  |
|  |  | TT | 27 |  |

Underlined values show statistical data with significant difference.
